# Integrating Genomics and Proteomics Data to Predict Drug Effects Using Binary Linear Programming

**DOI:** 10.1371/journal.pone.0102798

**Published:** 2014-07-18

**Authors:** Zhiwei Ji, Jing Su, Chenglin Liu, Hongyan Wang, Deshuang Huang, Xiaobo Zhou

**Affiliations:** 1 Division of Radiologic Sciences – Center for Bioinformatics and Systems Biology, Wake Forest School of Medicine, Medical Center Boulevard, Winston-Salem, North Carolina, United States of America; 2 School of Electronics and Information Engineering, Tongji University, Shanghai, P.R. China; University of Ulm, Germany

## Abstract

The Library of Integrated Network-Based Cellular Signatures (LINCS) project aims to create a network-based understanding of biology by cataloging changes in gene expression and signal transduction that occur when cells are exposed to a variety of perturbations. It is helpful for understanding cell pathways and facilitating drug discovery. Here, we developed a novel approach to infer cell-specific pathways and identify a compound's effects using gene expression and phosphoproteomics data under treatments with different compounds. Gene expression data were employed to infer potential targets of compounds and create a generic pathway map. Binary linear programming (BLP) was then developed to optimize the generic pathway topology based on the mid-stage signaling response of phosphorylation. To demonstrate effectiveness of this approach, we built a generic pathway map for the MCF7 breast cancer cell line and inferred the cell-specific pathways by BLP. The first group of 11 compounds was utilized to optimize the generic pathways, and then 4 compounds were used to identify effects based on the inferred cell-specific pathways. Cross-validation indicated that the cell-specific pathways reliably predicted a compound's effects. Finally, we applied BLP to re-optimize the cell-specific pathways to predict the effects of 4 compounds (trichostatin A, MS-275, staurosporine, and digoxigenin) according to compound-induced topological alterations. Trichostatin A and MS-275 (both HDAC inhibitors) inhibited the downstream pathway of HDAC1 and caused cell growth arrest via activation of p53 and p21; the effects of digoxigenin were totally opposite. Staurosporine blocked the cell cycle via p53 and p21, but also promoted cell growth via activated HDAC1 and its downstream pathway. Our approach was also applied to the PC3 prostate cancer cell line, and the cross-validation analysis showed very good accuracy in predicting effects of 4 compounds. In summary, our computational model can be used to elucidate potential mechanisms of a compound's efficacy.

## Introduction

The identification and functional understanding of a compound's effects on the pathway level is becoming more and more important [1]. It is a critical channel to deeply study the mechanisms of cancer cells so that more effective drugs can be developed. The Library of Integrated Network-Based Cellular Signatures (LINCS) project (http://www.lincsproject.org/) aims to create a network-based understanding of biology by cataloging changes in gene expression and other cellular process that occur when cells are exposed to a variety of perturbations. The gene expression data in LINCS (L1000) were cataloged for human cancer cells treated with compounds and genetic reagents. Similar to Connectivity Map (CMap) [2], the L1000 assay (Luminex-bead detection system) aims to connect diseases with genes and drugs at low costs. The gene expression profiles from L1000 data are potentially useful to infer the targets of compounds. However, little is known about how the downstream pathways of the inferred targets in signaling pathway are affected. P100 data in LINCS is one type of phosphoproteomics data which contains measurements of hundreds of proteins (roughly 700 proteins in our study) for the MCF7, PC3, and HL60 cell lines treated by 26 compounds. Immobilized metal affinity chromatography was used to reflect the response of cancer cells and the change of pathways as a result of treatments. P100 data potentially reveal the phospho-signaling groups of compounds when the signaling pathways come to a steady state after treatments.

A key question is how to integrate these two types of data to systematically infer the cell-specific pathways induced by those perturbations, and then to predict the compound's effects. Because the measurements in P100 data only cover one time point (6 hours after administration of the compounds), traditional pathway modeling with ordinary differential equations may not be suitable to handle such kind of mid-stage phosphoproteomics data [3]. Mitsos et al developed an integer linear programming approach to identify drug effects from phosphoproteomics data by discerning topological alterations in pathways [4]. However, the causal relationships for phosphorylation in the signal transduction process can be reflected in early responses, they are hard to capture later on. For example, phosphorylation of ERK ½ peaks under the stimulation of EGF and decreases within 1 hour [5]. Therefore, the challenge is how to infer the cell-specific pathways using data obtained from mid- or late-stage signaling responses.

**Figure 1:**
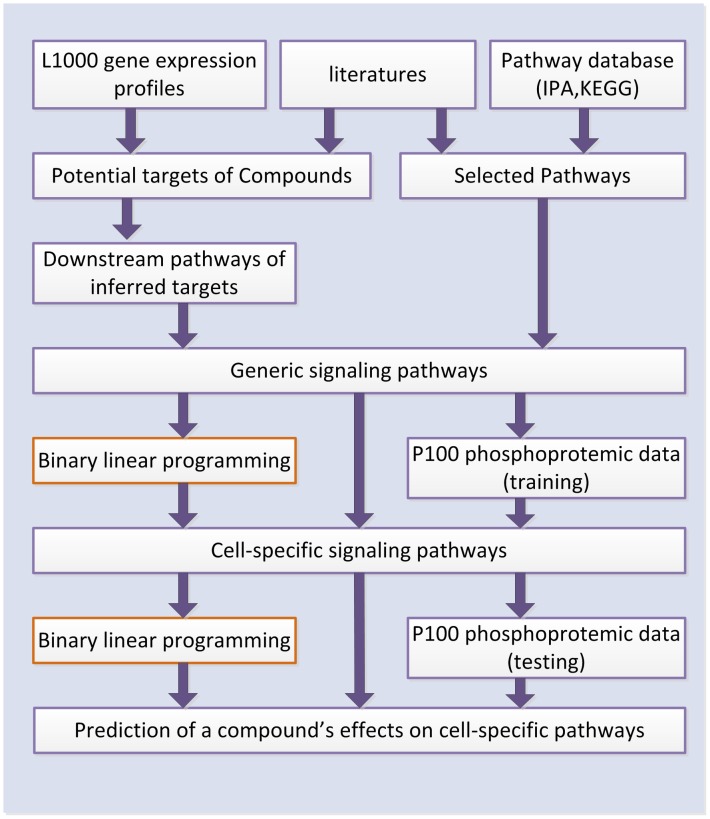
The flow chart of the proposed approach to infer a cell-type specific pathway map and to identify a compound's effects.

To address this challenge, we developed a binary linear programming (BLP) approach to predict a compound's efficacy by integrating L1000 gene expression and P100 phosphoproteomics data (**Figure 1**). In our approach, L1000 data are first employed to infer candidate targets of compounds and thereby create the generic pathway map. Secondly, we used BLP to optimize the generic pathways based on the mid-stage phospho-signaling response. Finally, we applied BLP to re-optimize the cell-specific pathways and thus evaluate the effects of compounds. To test the effectiveness of the proposed approach, we applied this approach to the MCF7 breast cancer cell line and the PC3 prostate cancer cell line. Cross-validation analysis showed that the cell-specific pathways inferred by our approach are reliable and the predicting accuracy of a compound's effects is high. In summary, our computational approach can shed light into the mechanisms of a compound's efficacy and facilitate drug discovery.

## Results

### Experimental data

In this study, we considered L1000 gene expression profiles and P100 phosphoproteomics data for MCF7 and PC3 cell lines (both included in L1000 and P100 data) treated with 15 compounds (**Table 1**). The 15 compounds were viewed as 15 sample conditions in the experimental data: The first 11 compounds were used to optimize the cell-specific pathways. The remaining 4 compounds were used to predict treatment effects (**Table 1**). We screened the gene expression profiles for 11 compounds and 3,712 shRNA-perturbations from the L1000 database to infer potential targets. For some compounds, their targets are unknown. These inferred targets were used as guides to create the generic pathway map (see **Materials and Methods**). We also screened two subsets from P100 phosphoproteomics data. The first subset with 11 compounds was employed to optimize the cell-specific pathways via our BLP approach. The second subset with 4 compounds was used to evaluate treatment effects by re-optimizing the inferred cell-specific pathways via BLP. The rationale is that we expect that some targets of certain compounds (e.g., Scriptaid [6]) were validated in literature and some targets of other compounds (e.g. daunorubicin) were unknown. Hence we need to infer the potential targets for those compounds with unknown targets based on the L1000 gene expression data. In the testing set, the first two compounds were HDAC1 inhibitors, while the targets of other two compounds were unknown. Based on the current fixed split, we tried to validate our model to learn whether the model could identify the effects of compounds even if their targets are unknown.

**Table 1:** The details of 15 compounds which are both covered in L1000 and P100.

Index	Compounds	Treatment Conc in P100 (uM)
1	Fulvestrant	1
2	Paclitaxel	1
3	Doxorubicin	6.8
4	GW-8510	10
5	Daunorubcin	7
6	Irinotecan	100
7	Scriptaid	10
8	Anisomycin	15
9	Valproic acid	1000
10	Digoxin	5.2
11	Geldnamycin	1
12	Trichostatin A	1
13	MS-275	10
14	Staurosporine	1
15	Digoxigenin	10.2

L1000 data were downloaded and processed as normalized log2 fold change value (http://cmap.github.io/l1ktools). The raw data of P100 (log2 ratio of treatment to control) were converted to binary values (0 or 1) according to the sign of raw data, where 1 corresponds to the fully activated state and 0 to no activation. In addition, if the targets or other co-regulators (some key proteins inhibited or activated after treatment) of some compounds were already validated in the literature, this prior knowledge was presented as constraints in our BLP approach.

### Proposed approach to infer cell-specific pathways and to identify a compound's effects

The workflow of the proposed approach is presented in **Figure 1**. In the first step, we inferred the potential targets of the compounds from L1000 gene expression profiles and information from the literature, and then created the corresponding downstream pathways of those inferred targets by integrating the PPI, transcriptional factor, and KEGG pathway database [7]. We also searched the pathways related to MCF7 and PC3 cell lines in IPA (http://www.ingenuity.com) and the literature [8]–[10]. After that, we integrated these pathways with inferred targets and their downstream pathways together to construct a generic pathway map. In the second step, the optimized cell-specific pathways were obtained by fitting the P100 data to the generic pathway map with our BLP approach. Finally, we applied BLP to re-optimize the cell-specific pathways to identify a compound's effects by discerning topological alterations in the pathway map.

### Inference of potential targets of compounds using L1000 gene expression profiles

To construct the generic pathway map, we needed to determine the potential targets of compounds in this study. If some targets were already validated in literature, they were directly included in our generic pathway map. For example, the compound Scriptaid's target is HDAC1, and thus HDAC1 is one protein included in our generic pathway map [6]. On the other hand, some compounds may not have validated targets, such as daunorubicin, digoxin, etc. If so, we inferred the targets from the L1000 assay. Here we briefly summarize target discovery using L1000 data. If a compound inhibits the function of a protein, the compound-induced L1000 gene expression changes should be similar to those of the knockdown of the gene of this protein. Therefore, the potential targets of a compound can be defined as a list of genes whose knockdowns show similar effects as this compound does on the gene expression.

We used an algorithm from Kolmogorov–Smirnov test-based gene set enrichment analysis (GSEA [11]) to calculate the Enrichment of Gene Effect to a Molecule. We assume that the disturbance of a gene by knockdown “drives” changes in expressions of other genes. Therefore, we defined each knocked-down gene as the “driver gene”. For each compound's effects on a cell line, enrichment of the compound-induced DEGs to the gene expression profiles induced by a knocked-down gene was calculated as a connectivity score (indicates the correlation between the compound's target and the driver gene). Top driver genes were screened as candidate targets according to their connectivity scores for a compound on that specific cell line. For example, STAT1 and HDAC1 were inferred as the potential targets of digoxin and irinotecan using the connectivity score, respectively. The details were described in **Text S1** in **File S1**.

Next, we created the corresponding downstream pathways of those targets by integrating the PPI data (HPRD [12]), transcriptional factor (TRANSFAC [13]), and KEGG pathway database [7]. PPI database also can provide some clues for pathway analysis because a certain protein might be regulated by its interaction partner via intracellular signaling [14], [15]. After inferring the targets of some compounds from L1000 genomics data, we started to search the paths connected with the targets based on the protein-protein interaction network. The paths will end at one protein when it is a transcriptional factor (TF). Finally, the directions of these paths were determined based on the knowledge from IPA, KEGG database, and literatures. For example, HDAC1 is a potential target of compound irinotecan and BRCA1 is a HDAC1's interaction partner, so that we have a potential path HDAC1 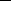 BRCA1. Meanwhile we also found another protein-protein-interaction partner of BRCA1 as P53 [16]; and then a potential downstream pathway of HDAC1 was determined as HDAC1 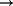 BRCA1 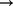 p53 [17], [18]. The results of inferred compound's targets and downstream pathways were represented in **Table S1** and **Table S2** in **File S1**.

### Construction of a generic pathway map

**Figure 2:**
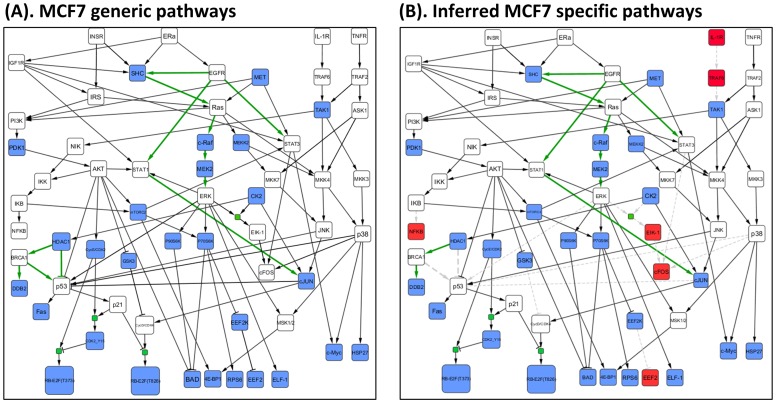
Boolean network topologies of the generic and inferred cell-specific pathways for the MCF7 cell line. (A) The MCF7 generic pathway map included some important classic pathways. The edges with green color were potential downstream pathways of some compounds. (B) After optimization via BLP, the red nodes and grey dash lines were removed from generic pathway map so that the cell-specific pathways were obtained.

After determining the potential targets of compounds and their downstream pathways, we manually created the generic pathway map. First, we searched the names of important pathways which were widely discussed in literatures; and then we used IPA to pick up the pathway topological structure of those pathways, including the estrogen signaling pathway, EGFR signaling, TNFR signaling, PI3K/AKT pathway, MEK/ERK pathway, JNK/p53/p21 pathway, p38 MAPK pathway, and HDAC1 pathway (**Figure 2A**).Because the pathway topology from IPA might not be suitable for all the cell lines, we combined the pathways from IPA with the potential downstream pathways of inferred targets together to get a generic pathway map. We show some important pathways in **Figure 2A**. The estrogen pathway induces tumor growth in estrogen receptor-positive breast cancers [6]; the PI3K/AKT pathway is an important player in cell survival [9]; the TNF signaling is anti-cancer related pathway [19]; and MEK/ERK pathways are usually associated with proliferation and anti-apoptosis; the NFkB pathway is involved in many cell functions, such as cell proliferation, cell survival, and cellular stress [20]; the JNK/p53/p21 pathway may induce cell apoptosis [8]; and HDAC1/BRCA1/DDB2 is a cell survival pathway [21]. In **Figure 2A**, the edges with green color are our inferred downstream pathways of some compounds. The results of inferred compound's targets and downstream pathways were represented in **Table S1** and **Table S2** in **File S1**.

**Figure 3:**
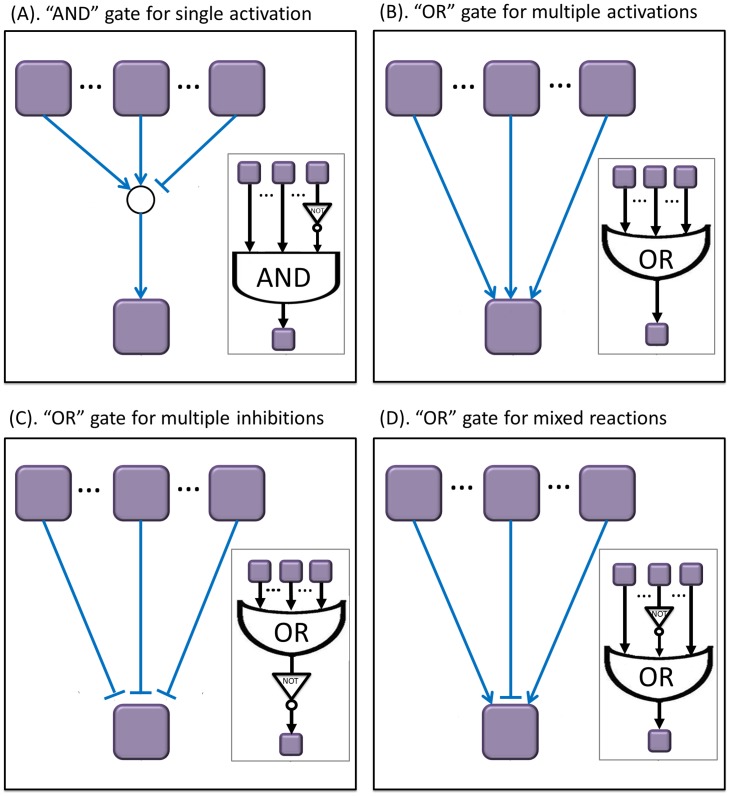
Four types of linking patterns in the pathway topological structures. Most of the actual pathways can be represented as Boolean networks using these linking patterns. (A) “and” gate for single activation; (B) “or” gate for multiple activations; (C) “or” gate for multiple inhibitions; (D) “or” gate for mixed reactions.

Next we represented the generic pathway map with Boolean operations (Boolean network) [22], [23]. Nodes in the network represent biological species and they have associated logical values (1 or 0) determining whether the species is activated or not. The signaling events (reactions) are encoded by Boolean operations on the nodes, which also have logical values (‘promotion’ (1) or ‘inhibition’ (0)). A Boolean network consists of a set of nodes and a set of directed edges. All the edges are linked via “OR” and “AND” logic gates. **Figure 2A** shows the Boolean topology of generic pathways for the MCF7 cell line. The actual signaling pathway map has many types of topological structures (shown in IPA and **Figure 2A**), but most signaling pathways can be summarized as a Boolean network using four topological structures (**Figure 3**). The two cases in **Figure 3A** (“AND” gate for single activation) and **Figure 3B** (“OR” gate for multiple activations) were discussed by Mitsos et al [4]. The topological structure pattern in **Figure 3A** indicates that a single activation will occur if no inhibitors are activated and at least one of signaling species is activated. **Figure 3B** shows that multiple activations can possibly activate the downstream protein and these activations are logic “OR”. However, these two linking patterns do not present all connections in pathway topology because of the complicated pathways in KEGG and IPA database (such as two cases in **Figure S5** in **File S1**). In the current study, we also considered two more cases in **Figure 3C** (“OR” gate for multiple inhibitions) and **Figure 3D** (“OR” gate for mixed reactions). **Figure 3C** suggests that multiple inhibitions can possibly inhibit the downstream proteins and these reactions are logic “OR”. As to Figure 3D, it indicates the states of downstream protein might be regulated by activation and inhibition, simultaneously. More details about the difference between our approach and the approach developed by Mitsos were described in **Text S3** and **Figure S6** in **File S1**. **Table 2** shows some combinations of our binary linear constraints developed to infer the states of nodes and edges from these topological structures. Details regarding these constraints in **Table 2** were described in **Text S2** in **File S1**. For more details regarding Boolean representation of pathways, please refer to [22].

**Table 2:** Four types of linking patterns between species and products in pathway topology.

Type	Logic gate	The index of Constraints for addressing each type of topological structure	Description
A	AND gate	(7),(8),(9),(10),(11)	Single activation
B	OR gate	(7),(9),(10),(11)	Multiple activations
C	OR gate	(7),(9),(12),(13)	Multiple inhibitions
D	OR gate	activation: (7),(13),(14) (15)	Mixed reactions
		inhibition: (7),(13),(12) (15)	

### Inference of cell-specific pathways by BLP

To infer cell-specific pathways based on the generic pathway map constructed above, we then minimized the differences between the measurements and the simulated values, as well as the complexity of the signaling pathway structure's topology. To simplify this optimization problem, we developed a binary linear programming (BLP) approach to optimize such multi-objective functions. The concept behind BLP is that the states of the proteins (variables) are normalized to binary numbers (activated state or no activation); edges between two proteins are also represented as binary numbers (inhibition or promotion); binary linear constraints are used to describe the relationship between upstream and downstream proteins; and the optimization is done with binarized values taken by variables, edges, and constraints [4], [24](see **Materials and Methods**).

The BLP is solved with the optimization toolbox in MATLAB that guarantees minimal differences between phosphoproteomics data and predicted data, as well as the Boolean topology of the generic pathway map. Because P100 data are captured at the mid-stage of signal transduction, we developed constraints to simulate the change from early to mid-stage so that we can still obtain many important causal relationships of phosphorylation. The fitting precision of the optimized cell-specific pathways is 87.66% (**File S2**) for MCF7, which proves that our model works well on mid-stage phosphoproteomics data.

**Figure 2B** shows the inferred cell-specific pathways of MCF7. The blue nodes are the measured phosphoproteins, while the red nodes and grey dotted lines were removed after optimization. After our BLP system simultaneously optimizes the two objective functions with the P100 data, we keep those reactions whose edges exist for some compounds, and remove those reactions whose edges completely do not exist for all compounds. For example, HDAC1 inhibitor-induced JNK activation in turn activates the downstream pathways p53 and p21 (JNK 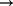 p53 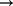 p21), and eventually results in cell cycle arrest. Another example is that the reaction between cJUN and p53 (cJUN decreases the expression of p53) did not appear in the pathways induced by all compounds, so we removed this reaction, although it does exist for certain conditions [25]. In addition, to keep the proteins at the end of each pathway as measured phosphoproteins, all other proteins at the end of each pathway not measured in P100 were removed (e.g. (ERK AND CK2) 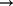 ELK-1 reaction in **Figure 2B**). Thus, the inferred cell-specific pathway map was smaller but contains only those elements that can fit the experimental evidence very well. When this approach was applied to the PC3 cell line, the goodness of data-fitting on the inferred cell-specific pathways was 90.91%. The details of the generic and inferred specific pathways of PC3 are shown in **Figure S1** in **File S1**. In addition, the effects of the different combinations of compounds to the inferred cell-specific pathway map were described in the section of “Discussion”.

### Cross-validation

To prove the reliability of the developed approach, we examined the effects of leave-one-out cross-validation on the phosphoproteomics data of 11 compounds. For each compound, we employed data from 10 compounds to optimize a cell-specific pathway topology and evaluate the treatment effects of the remaining one compound on this pathway map by predicting the states of all the proteins via BLP. The mean values for fitting precision in MCF7 and PC3 cell lines were 84.85% and 86.20%, respectively, which suggests the inferred cell-specific pathway map is reliable for prediction of a compound's effects (**File S3**). According to the leave-one-out cross-validation we did on MCF7 cell line, we calculated the similarity of topological structures among 11 cases (each corresponds to one compound) where each case represents cell-specific pathway map trained from the remaining 10 compounds after leaving one compound out). The connected edges in all inferred pathways are 80. The similarity of the inferred cell-specific pathways based on the different combinations is defined as the ratio of the number of connected edges in each cell-specific pathway divided by the total number of connected edges (80). The average value of the similarity of 11 cell-specific pathways generated by leave-one-out cross-validation was 98.9%. This again indicates our inferred cell-specific pathway map was reliable.

### Prediction of a compound's effects via compound-induced topological alterations

**Figure 4:**
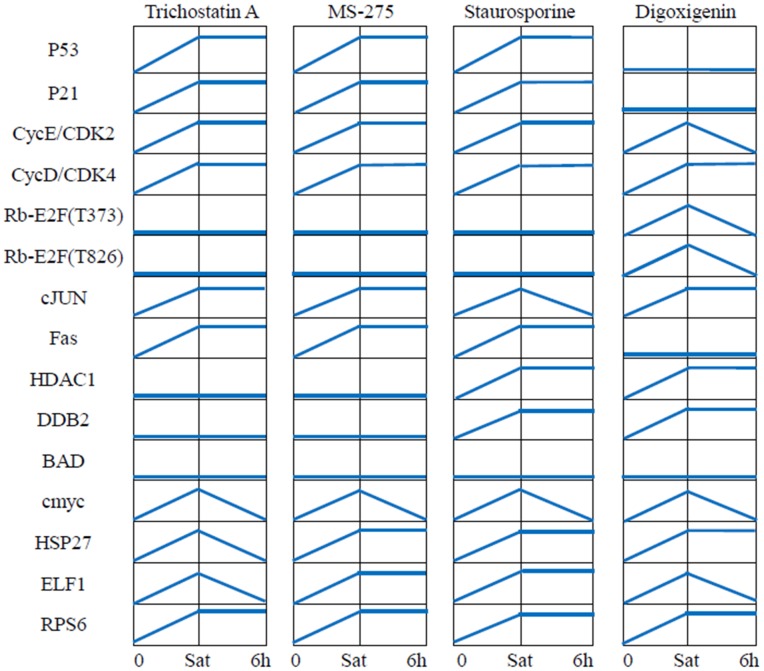
Change of states of some important proteins during two time points. In this figure, the binary value “1” and “0” are represented as high level and low level signal, respectively. Sat means the moment that the signaling pathways come to saturation condition in the early stage of signal transduction after treatment with compound. In the BLP approach, Sat and 6 h are the time 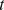 and 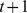, respectively.

**Figure 5:**
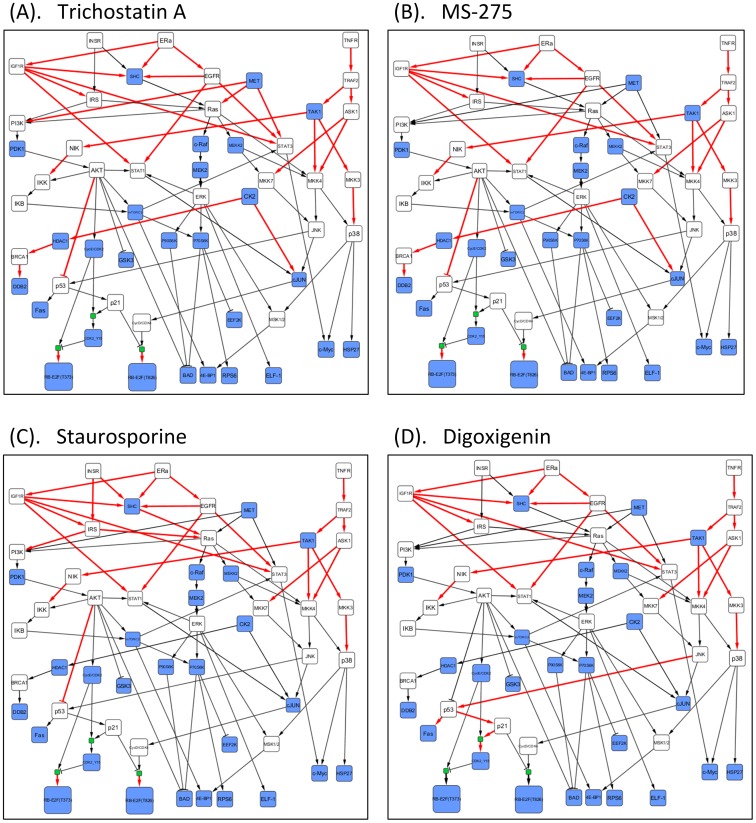
The BLP approach revealed the compound-induced topological alterations in the MCF7-specific pathways. The treatment effects of four compounds on MCF7 cell line were shown in the Figure. Red arrows denote these reactions were blocked after treatment with compounds.

Our assumption is that the cell-specific pathways are fixed when there is no perturbation. So we can try to infer the cell-specific pathways first with some drug treated data and then use the reconstructed cell-specific pathways to predict effects of new drugs. Here, we first used the BLP model to train the generic pathways with the first part of phosphorylation data (11 training compounds), and obtained the cell-specific pathways. Then we fit the second part of data (4 testing compounds) to the optimized cell-specific pathways with BLP model, the state of phosphoproteins could be inferred to represent the compound's treatment effects via topological alterations. **Figure 4** shows the changes of states at 0 min, saturation state, and 6 hour of some important phosphoproteins in the downstream of the signaling pathway map where 12 proteins are measured in P100 (3 proteins are not). These predicted changes of states are consistent with the biological functions shown in **Figure 5**. In addition, for all 27 measured phosphoproteins in the MCF7-specific pathways, the accuracy of data-fitting of these phosphoproteins for 4 compounds were 85.19%, 81.48%, 92.59%, and 96.30%, respectively (**File S2**). The detailed results were discussed below.

For identification of a compound's effects, we applied the inferred MCF7-specific pathways to 4 compounds: trichostatin A, MS-275, staurosporine, and digoxigenin. The first two compounds are HDAC inhibitors [26], [27], and staurosporine promotes apoptosis [10]. **Figure 5** depicts the pathway's topological alterations for these compounds. Including trichostatin A, a HDAC inhibitor, removes the branches (subsets of the pathways) as follows: some downstream paths of IGF1R and EGFR, TNFR 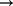 TRAF2 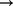 TAK1, AKT 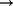 p53, HDAC1 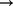 BRAC1 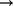 DDB2, CycE/CDK2 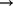 Rb-E2F (phosphosite Thr373) and CycD/CDK4 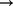 Rb-E2F (phosphosite Thr826). **Figure 5A** suggests that HDAC1 and its downstream pathway are blocked, which may cause cell growth arrest. The p53 signal is up-regulated after the activation of JNK. This was also confirmed in **Figure 4**, where the states of the phosphoproteins were inferred with BLP. In the meantime, p21 was activated by p53, which then induced cell cycle arrest by inhibiting phosphorylation of the Rb-E2F complex triggered from CycE/CDK2 and CycD/CDK4 [28]–[30]. In addition, up-regulated Fas activated by p53 will potentially promote cell apoptosis.

MS-275 [27], also a specific HDAC inhibitor, altered the pathway topology in a similar pattern as trichostatin A. MET was inhibited by trichostatin A but activated by MS-275 (**Figures 5A and 5B**). These two compounds induced similar changes on most key proteins; only the expression of HSP27 and ELF1 were different (**Figure 4**). In **Figure 5B**, p21 activation inhibited phosphorylation of the Rb-E2F complex and blocked the disassociation of this complex [28]–[30]. Therefore, our results suggest that the effects of trichostatin A and MS-275 block cell growth in the MCF7 cell line.

With regard to staurosporine [10], a pro-apoptotic compound, double effects were detected from the pathway's topological alterations: p21 obviously blocked the cell cycle by inhibiting the phosphorylation of the Rb-E2F complex, while DDB2-induced cell growth also occurred via activated HDAC1 (**Figure 5C**). **Figure 4** also shows similar results.

Digoxigenin induced the activation of HDAC1 and inhibition of p53 (**Figure 5D**). It blocked the reaction JNK 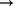 p53 so that p21 was also inactivated. DDB2 was activated by HDAC1 through BRCA1. The absence of p21 indicates phosphorylation of the Rb-E2F complex, which increases the chance for the disassociated transcription factor E2F to promote transcription and cell growth [30]. Therefore, our findings indicate digoxigenin potentially induce cell cycle and promote cell growth on MCF7 cell line.

As shown in **Figure S2** in **File S1**, MS-275, staurosporine, and digoxigenin induced the inhibition of TNFR and part of its downstream signaling pathway such as IKK, which is very important for survival of PC3 cells [31]. Finally, double effects were detected: p21 blocked the cell cycle, but HDAC1 induced cell growth via DDB2 (**Figure S2C** and **S2D** in **File S1**).

## Discussion

In this paper, we present a computational approach to optimize generic pathways and identify a compound's effects on an inferred cell-specific pathway map by integrating gene expression profiles and phosphoproteomics data collected from various types of perturbations. For constructing the generic pathways, we combined the pathway information from the literature and the potential targets of compounds inferred from gene expression profiles under perturbations. A generic pathway map of MCF7 cell line with 60 proteins and 94 reactions was obtained. With our binary linear programming (BLP) approach for pathway topology optimization, a cell-specific pathway map of 54 proteins and 80 reactions that cover 27 measured phosphoproteins was finally inferred. In the optimized cell-specific pathways, we monitored 4 cases of compound-induced topological alterations to the pathways to predict a compound's effects using BLP.

We used BLP formulations in two ways. For inference of the cell-specific pathways using the perturbation-induced data of 11 compounds, the BLP system included 4,014 constraints and 2,002 variables in the generic pathways. When the optimized cell-specific pathway map was utilized to predict the effects of each compound, it had about 306 constraints and 155 variables every time. In addition, there were 27,887 constraints and 9,732 variables used in a generic pathway map of 74 proteins and 105 reactions in ILP approach [4]. Then 2,477 constraints and 947 variables were needed for predicting the effects of each compound on the cell-specific pathway map, which contained 49 proteins and 44 reactions. These data suggest our BLP approach is significantly simplified and less redundant than that in [4].

Compared to other phosphoproteomics-based and mass spectrometry-based target identification approaches, which use compound affinities measured either by *in vitro* or *in vivo* assays, our method uses perturbation-induced gene expression profiles to infer the potential targets and downstream paths [32], [33]. After that, a generic pathway map could be created based on pathways in the literature and inferred targets. We developed BLP to monitor alterations in pathway topology as a way to evaluate a compound's effects.

An important aspect of our approach is the inference of the cell-specific pathways. Our approach, which is based on Boolean modeling, can simplify the representation of pathway topology and boost the optimization process. Compared with ordinary differential equation-based methods, a Boolean logic model has limited abilities to model kinetic behavior, but as a non-parametric estimation approach it can be applied for the simulation of large-scale topological structures. The actual signaling pathways have many types of topological structures, but the ILP approach can only deal with two examples of the topological structure, and its rules are rather redundant [4]. Our method can handle four types of linking patterns of topological structures in pathway map. Our binary linear constraint system can infer the cell-specific pathways from the mid-stage phospho-signaling response, which only covers one time point, by constructing rules that reflect the continuous changes of phosphorylation or states. In addition, we simplified the design of constraints, so that our Boolean model contains much fewer constraints than others [4].

### Effects of Binarization on resulting networks

We also notice that different binarization techniques resulted different networks. We investigated the effects of three binarization approaches on our resulting networks [24]. In our study, the P100 phosphoproteomics data is defined as log2(treatment/control). We used zero (“0”) as a fixed threshold. If the state of a protein is positive (>0), it indicates this protein is up-regulated; otherwise, this protein is down-regulated. The BASC approach tries to find a robust threshold for data binarization by assessing whether such a threshold is possible to divide the data into two stable groups at different scales [24]. The basic idea is to build a family of 1-dementional time series by approximating the original ordered time series gene expression data with step functions, whose number of discontinuities decreases gradually. The authors proposed two algorithms (BASC A and BASC B) for implementing this idea. BASC A performs better than BASC B without elimination of noisy genes. Hence, we applied BASC A approach to our MCF7 phosphoproteomics data. The **Figure S7** in **File S1** presents the MCF7-specific pathway network inferred using BASC A approach. After comparing with the pathway network inferred using our normalization method in **Figure 2B**, we found that these two cell-specific pathway networks were identical except an extra link (HDAC1 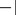 p53) found by BASC A. Biologically HDAC1 can increase inhibition of phosphorylated active p53 [34]. In addition, the fitting precision of our method is also similar to BASC A (**Figure S8** in **File S1**). In conclusion, BASC algorithm also can be applied to binarize p100 data. Some detailed results about BASC approach applied to our MCF7 data are presented in **Text S4** in **File S1**. In addition, we also selected two well-defined binarization techniques on MCF7 phosphoproteomics data: (A) we used the mean value as a threshold [35] (B) we used a K-means clustering approach to find a threshold [36]. Based on the first method, the inferred MCF7-specific pathway topology included 80 edges. The fitting precision of the inferred cell-specific pathways to 11 compounds is 87.99%. When the cross validation was applied on the 11 compounds, the fitting precision is 85.5%. We then used the inferred specific pathway network to identify the effects of 4 compounds. The average prediction accuracy is 87.04%. Moreover, for the second method, our inferred MCF-7 specific pathway topology included 79 edges. The fitting precision of the inferred cell-specific pathways on 11 training compounds is 88.31%. The average predicting accuracy on 4 testing compounds is 87.96%.

When applied to the MCF7 cell line, our approach identified both known and unanticipated results. Trichostatin A and MS-275 both are HDAC inhibitors; thus, their inhibition of HDAC1 can be seen in the pathway map and cell cycle is blocked via the up-regulated p53. Digoxigenin acts on MCF7 cells to inhibit p53 and induce cell cycle changes and cell growth promotion. In the case of staurosporine, a pro-apoptotic compound, double effects were detected from the pathway's topological alterations: p21 blocked the cell cycle by inhibiting the phosphorylation of the Rb-E2F complex, and DDB2-induced cell growth occurred via activated HDAC1. Finally, we detected no obvious topological alterations for the proteins BAD, 4E-BP1, RPS6, or c-Myc (**Figure 5**). It is possible that our mid-stage phosphoproteomics data missed some early response signals, so that the proposed approach might not detect all the changes of reactions.

When we applied the proposed approach to the PC3 cell line, the precision of fit for the inferred cell-specific pathways on the experimental data of 11 compounds was 90.91% (**File S2**). We also evaluated the effects of 4 compounds (trichostatin A, MS-275, staurosporine, and digoxigenin) on the PC3 cell line. All these compounds induced the inhibition of EGFR, which plays an important role in apoptosis [37]. Comparing the four subfigures in **Figure S2** in **File S1**, MS-275, staurosporine and digoxigenin induced the inhibition of TNFR and part of its downstream proteins such as IKK which is very important for cell survival of PC3 [31]. Finally, double effects were detected in **Figure S2C** and **S2D** in **File S1**: p21 blocked cell cycle; however, HDAC1 induced cell growth via DDB2.

### The effects of different combinations of compounds

We tried to test the changes of MCF7-specific pathways by using different combinations of compounds from 5, 6, …, to 15. For example, according to the order shown in **Table 2**, we tried to combine the first five compounds for inferring the cell-specific pathways; then we added the sixth compound to that pool to infer the cell-specific pathway again, …, we repeat this procedure until the combination includes all the 15 compounds. The connected edges in all inferred pathways are 80. The similarity of the inferred cell-specific pathways based on the different combinations is defined as the ratio of the number of connected edges in each cell-specific pathway divided by the total number of connected edges (80). We notice that the different combinations with 5 to 9 training compounds have the similarity value being 88%. However, the similarity is 100% for the cases trained with the combination of compounds from 10 to 15. This result indicates that the inferred topological structure will be steady when at least 10 training compounds were used.

### Redundancy analysis

In our study, the redundancy analysis (RDA) of compounds was performed on our P100 dataset via R-package “Vegan” [38]. In **Figure S4** in **File S1**, the correlations among 11 training compounds in the training set were represented with blue arrows and 4 testing compounds were denoted with red markers. The cosine of the angle between blue lines is approximately equal to the correlation between the corresponding variables. “sit1”-“sit28” means the markers of 28 measured proteins in the generic pathway map. Obviously, most of the blue lines (training set) have obvious angles; the average value of the correlation coefficient between any two training compounds is 0.31. Therefore, there was low redundancy of these 11 compounds. Based on the result shown in **Figure S4** in **File S1**, we considered our LOO cross-validation on training set as reliable.

### Network behavior

Although network behavior is not the focus of this study, we tried to evaluate our network behavior over time after a drug is applied based on the inferred pathway topology when most proteins researched to saturation conditions through phosphorylation. Because the state of most proteins (in cell-specific pathways) at time 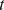 (defined in BLP model, see the section of **Materials and Methods**) satisfied our Boolean constrains, cell-specific pathway network treated by certain compound transits from saturation state to a steady state (all the proteins had no-changes) more quickly than this network transits from a random state. As an example with MCF7 cell line, we inferred the states of all the proteins at next time point (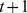) to represent the next state of the pathway network. The states of a downstream protein at 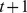 were inferred from the states of its upstream proteins at time 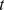 with constraints and pathway topological information. By that analogy, we can infer the transition of the network's states from the saturation condition (time point 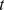) to the steady state. Treated by 11 training compounds separately, the saturation state of MCF7-specific pathway network reached to a steady state with 7.1818 steps by average; when we randomly generated 10 (random) states of the specific pathway network, it were 10.3 steps for the network to reach a steady state.

In summary, we provide a novel methodology to infer the cell-specific signaling pathways and to identify the effects of compounds on the pathways. Our BLP approach can quickly infer the best-fitting pathways from topological structures calculated from the perturbation-induced data. We believe the proposed approach complements standard biochemical drug profiling assays and sheds new light on the discovery of possible mechanisms for drug effects.

## Materials and Methods

### Experimental data

In the LINCS project, L1000 gene expression profiles and P100 phosphoproteomics data have been generated by the Broad Institute (http://lincscloud.org/exploring-the-data/data-api/). The L1000 database is a catalog of gene-expression profiles collected from human cells treated with compounds and genetic reagents. These data are used to reveal connections between genes and compounds and the related molecular pathways for underlining disease states. The L1000 database includes information on 5,178 small-molecule compounds; 3,712 genes perturbed using lentivirally-delivered shRNAs; and the effects of overexpression of those genes. All the data were collected from 15 cancer cell lines on 1,000 carefully chosen landmark genes, which can reduce the number of measurements and will not be biased for a particular cellular model. The data from the 1,000 landmark genes will be converted to expression data for about 22,000 genes (http://lincscloud.org/the-data-set/the-landmark-genes/)

The P100 database contains measurements of phosphorylation caused by 26 different compounds for three cell lines (MCF7, PC3, and HL60). The treatments were each administered for 6 hours at 37°C and were repeated twice in complete biological replicates. Details of the experimental design can be found at (http://lincscloud.org/exploring-the-data/data-api/phosphoproteomics). Treated cells were grown in SILAC medium [Arg-6, Lys-4] or heavy [Arg-10, Lys-6] growth medium; control cells treated with DMSO were grown in light [Arg-0, Lys-0] growth medium. All raw values in P100 represent log2 ratios of the treatment vs. DMSO.

### Computational procedure: Binary linear programming

Here, we describe how the Boolean model can be reformulated as a BLP to optimize the cell-specific pathways. Two reports in the literature [4], [22] used a Boolean model to optimize the generic pathway map under the stimulation of combined different cytokines. However, their models are designed only for phosphoproteomics data in the early stage of signal transduction. Although the causal relationships for phosphorylation could be reflected from the early response of signal transduction, they are hard to capture after the mid-stage of the response. Therefore, these models might be unsuitable to optimize pathway maps by using mid- or late-stage phosphoproteomics data directly for some phospho-signaling response that occurs only in the early stage. For inferring a cell-specific pathway map using the P100 database with only one time point (6 hours), we assume a virtual time point (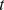) before 6 hours, which represents most of enzyme's activities reach to saturation conditions after phosphorylation at time 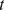. The cell- specific pathways inferred by our BLP approach corresponds to the topological structure in the saturation condition in early response. The observed time point (6 hours) could be represented as 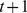, indicating the mid-stage signaling response. In our BLP approach, we employ binary variables to describe the phosphorylation states of enzymes and the reactions (activated or inhibited). We also use binary linear constraints to model the relationship between the early response at 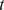 and mid-stage response at 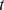 +1. According to the concept of Hill Function [39], there are three scenarios for the state of enzyme 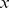 at time 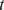 and 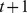 in our BLP approach:

(A) Eq. (1) suggests that 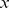 is activated by its upstream enzyme in the early stage, reaches a steady state at time 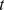, and its activity is unchanged until time 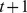.
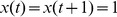
(1)


(B) If the state of enzyme 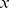 at time 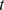 and 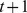 can be present as Eq. (2), 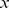 is activated in the early stage (its activity reaches steady state), and then enzyme 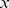 is gradually degraded so that its activity is very low at time 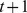.
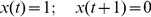
(2)


(C) When treated with a compound, enzyme 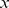 is inhibited at time 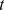, and its activity will be sustained to time 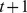 (Eq. (3)). 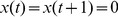
(3)


Similar to two previous reports [4] and [22], the states of all proteins at time 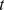 will completely satisfy the causal relationships in our constraint set. The change of states for each measured phosphoprotein is also considered between two time points.

A pathway is defined as a set of reactions 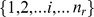 and species 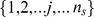. Each reaction has three corresponding index sets, signaling molecules 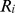, inhibitors 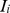, and products 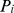. These sets are subsets of the species index set (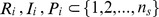). Compared to [4], our binary linear constraint system can address four types of linking patterns to represent the relationships between species and reactions, since an actual signaling pathway has many types of topological structures (**Text S2** in **File S1**). Due to the different mechanisms of compounds, a reaction will take place after treatments with some compounds but not others. The goal of the proposed formulation is to remove the redundant and inconsistent reactions which do not occur with any compounds.

For a generic pathway map, a set of experiments (indexed as 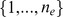) can be performed. Each experiment indicates a treatment condition of one compound on the pathway. In the 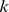-th experiment (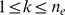), a subset of species is activated and another subset is inhibited, summarized by the index sets 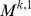 and 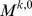, respectively. In addition, a third subset of the species is measured around the phosphorylation level (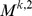). In our BLP system, a binary variable 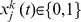 indicates if the species 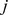 is activated (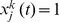) or not (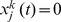) at the time point 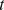 in the 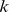-th experiment. The variable 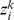 indicates if the reaction 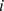 takes place (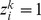) or not (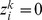) in the 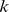-th experiment. The species set 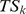 denotes the potential targets of 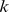-th compound.

If the phosphorylation level of species 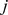 is measured at time 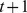 in P100 data, the measurement of species 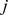 is defined as 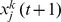 and its predicted value is named as 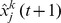. For inferring the cell-specific pathways, we use our BLP approach to optimize two objective functions. The first is to minimize the difference between predicted values and measurements (Eq. (4)): 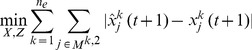
(4)


The second objective 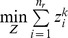 is to minimize the number of reactions, so that the scale of the inferred cell-specific pathways is smaller. The Eq. (4) is equivalent to Eq. (5) (see **Text S2** in **File S1**).
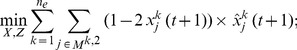
(5)


Here, we use a linear solution to simultaneously optimize the two objectives above: 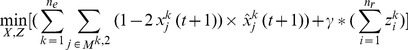
(6)


In Eq. (6), the parameter 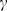 indicates the weight between two objective functions. The selection of values of 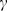 obviously affects the fitting precision of our model on experimental data (**Figure S3** in **File S1**).

The constraints in our BLP approach can be summarized as: 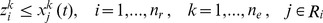
(7)




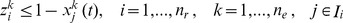
(8)




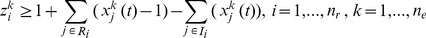
(9)




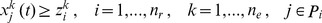
(10)

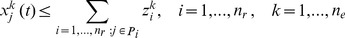
(11)

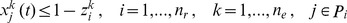
(12)

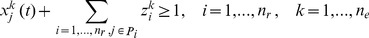
(13)

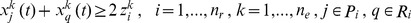
(14)

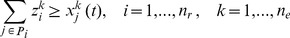
(15)

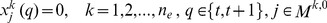
(16)

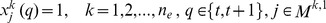
(17)

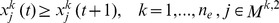
(18)

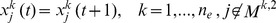
(19)

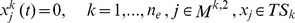
(20)

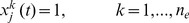
(21)

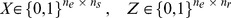
(22)


According to these formulas, the constraints (7)–(15) in our BLP system are used to infer the states of all the species and reactions according to the pathway's topological structure. These constraints can handle four types of linking patterns between species in the pathway map. The detailed explanations of constraints (7)–(15) are described in the **Text S2** in **File S1**. The states of all variables in 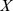 (all the proteins) should meet the constraints (16) and (17). The constraints (18) and (19) simulate the change of a phosphoprotein's state from time point 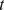 to 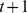. If the phosphorylation level of a species is measured at time point 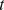, we assume its activity may be consistent or degraded (constraint (18)). If the species is not measured, we keep its activity unchanged during two time points because there is no available information about it (constraint (19)). The constraint (20) set the states of some potential target proteins which are validated in literature. The constraint (21) set the states of some important activated proteins which are validated in literature. The formula (22) restricts the values of two groups of binary variable 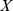 and 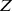.

### Computational procedure: fitting precision of data

For the fitting precision (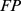), we calculated the percentage of fit (prediction accuracy) as below:
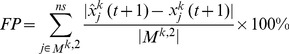
(23)


Eq. (23) indicates the fitting precision between the predicted values inferred by our BLP approach and the measured values of species under the treatment of 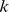-th compound, where 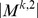 is the number of species in the set 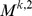. The value of fitting precision (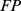) is in the range between 0 and 1.

## Supporting Information

File S1 **The supplementary materials, including Text S1-S4, Figure S1-S8, and Table S1-S2.**
(DOCX)

File S2 **The fitting precision of training compounds and testing compounds in two cell lines.** (XLSX)
(XLSX)

File S3 **The fitting precision of cross validation in two cell lines.** (XLSX)
(XLSX)
