# Supplementary material for: Integrating Genomics and Proteomics Data to Predict Drug Effects Using Binary Linear Programming
Source: PLoS One. 2014 Jul 18;9(7):e102798. doi: 10.1371/journal.pone.0102798 (PMC4103865; doi:10.1371/journal.pone.0102798)
Supplement: File S1 — The supplementary materials, including Text S1-S4, Figure S1-S8, and Table S1-S2. (DOCX) [file pone.0102798.s001.docx]

**Integrating** **genomics and proteomics data to predict drug effects**

**using Binary Linear Programming**

Zhiwei Ji**^1, 2^**, Jing Su**^1^**, Chenglin Liu**^1^ ,** Hongyan Wang**^1^**, Deshuang Huang**^2^**, Xiaobo Zhou**^1,*^**

**1** Division of Radiologic Sciences – Center for Bioinformatics and Systems Biology, Wake Forest School of Medicine, Medical Center Boulevard, Winston-Salem, NC, USA 27157

**2** School of Electronics and Information Engineering, Tongji University, Shanghai, P.R. China 201804

^*^ Corresponding author: Department of Radiology, Wake Forest School of Medicine, One Medical Center Boulevard, Winston-Salem, NC, USA 27157. Tel.: +1 336 713 1879; Email: [xizhou@wakehealth.edu](mailto:xizhou@wakehealth.edu).

**Supporting information**

**Table of Contents**

**Text S1** Enrichment of gene effect to molecule for drug target discovery.

**Text S2** The description of developed binary linear programming (BLP) approach.

**Text S3**  The difference between BLP approach and other approach.

**Text S4** The effects of BASC normalization algorithm on resulting networks.

**Figure S1**  The generic pathways and inferred specific pathways of PC3 cell line.

**Figure S2**  Compound-induced pathway topological alterations on PC3 cell line.

**Figure S3** The fitting precision curves of the cell-specific pathways.

**Figure S4**  The result of redundancy analysis on P100 data with R-package “Vegan”.

**Figure S5**  Two cases about two linking patterns which are mentioned in Figure 3C-3D.

**Figure S6**  A case about multiple activations is used to describe how the Boolean function can be built depends on topological structure.

**Figure S7** The inferred MCF7-specific pathway map based on the P100 data which was binarlized with

BASC_A algorithm. The topology of this pathway map is similar to Figure 2B.

**Figure S8**  The performance of BASC A approach and our method.

**Table S1** The inferred potential targets of some compounds.

**Table S2** The potential downstream pathways of inferred targets included in the generic pathways.

**References**

**Supplementary text**

**1. Enrichment of Gene Effect to a Molecule for drug target discovery**

If a compound inhibits the function of a protein, the compound-induced gene expression changes should be similar to that of the knockdown of the gene of this protein. Therefore, the potential targets of a compound can be defined as a list of genes whose knockdowns show similar effects as this compound does on the gene expressions. We use a Kolmogorov–Smirnov test based gene set enrichment analysis (GSEA) [[1](#_ENREF_1)] algorithm to calculate the Enrichment of Gene Effect to a Molecule. We assume that the disturbance of a knocked-down gene “drives” the changes of the expressions of other genes. Hence, we defined each knocked-down gene as the “driver gene”. For each compound treated on a cell line, the enrichment of the compound-induced DEGs to the gene expression profiles induced by a driver gene was calculated as connectivity score (indicates the correlation between the target of compound and the driver gene). Top-ranking driver genes are screened as candidate targets according to their connectivity scores for a compound on that cell line. The computational steps are following:

1. Pick up the DEGs for each compound’s treatment.

Given a threshold (P-value=0.05), the R-package Limma is used to select a differential expression gene set $D_{i} =\{g_{1} ,g_{2} ,...,g_{m} \}$ from the gene expression profiles under the treatment of compound $c_{i}$, where $m$is the number of DEGs in $D_{i}$. And then $D_{i}$ is partitioned to up- and down- regulated gene subsets as $dsub_{up}^{i}$ and $dsub_{down}^{i}$.

1. Calculate the connectivity score of DEGs $D_{i}$ .

We assess the enrichment of $D_{i}$ to each gene-expression signature of the shRNA-perturbation via one driver gene. To do that, the connectivity score $cs_{i}^{k}$of $D_{i}$is calculated by GSEA with a reference gene-expression signature $R_{k}$, which is obtained by knocking down the $k$-th driver gene. After ordering the signatures $dsub_{up}^{i}$,$dsub_{down}^{i}$, and $R_{k}$, we calculate the connectivity score $cs_{i}^{k}$ as following equations with the notation used in R package:

(1)

$p=\max\{s_{i}^{1} ,s_{i}^{2} ,..s_{i}^{k} .,s_{i}^{n} \}$ (2)

$q=\min\{s_{i}^{1} ,s_{i}^{2} ,..s_{i}^{k} .,s_{i}^{n} \}$ (3)

$cs_{i}^{k} =\left\{\begin{matrix} \frac{s_{i}^{k} }{p} if\mathop{}\limits s_{i}^{k} >0 \\ \frac{-s_{i}^{k} }{q} if s_{i}^{k} <0 \end{matrix}\right.$ (4)

The above formulations indicate the score $cs_{i}^{k}$ is ranged between -1 and 1. When $cs_{i}^{k}$ is equal to 1, that means knocking down of the inferred target (driver gene) has the same effect with the compound’s treatment on the actual target, hence, we can view the inferred target as a candidate target or a positive co-regulator of the actual target. When $cs_{i}^{k}$ is equal to -1, that indicates the inferred target (driver gene) and actual target have inverse transcriptional effects to the downstream pathways after the treatment of compound $c_{i}$, therefore, we consider our inferred target may be located closely to the actual target and there may be one negative regulatory relationship between them. For example, STAT1 and HDAC1 were the potential targets of compound digoxin and irinotecan, respectively (**Table S1**). The connectivity score of STAT1 equals to 0.93775, which means knocking down of STAT1 induced very similar transcriptional effect as treatment with compound digoxin. Hence, we considered STAT1 was a potential target of digoxin. However, the connectivity score of HDAC1 (-0.97890) indicates there might be a reversible regulatory connection between HDAC1 and the actual target of irinoteacn.

1. Select the potential target of compound.

A connectivity score list $cslt_{i} =\{cs_{i}^{1} ,cs_{i}^{2} ,...,.cs_{i}^{k} ,..,cs_{i}^{n} \}$ can be calculated for the compound $c_{i}$, where $cs_{i}^{k}$ is the connectivity score between compound $c_{i}$ and $k$-th perturbed gene. We set a threshold value to pick up several top-ranking potential targets from $cslt_{i}$. In our experiment, we set $\left|s_{i}^{kj} \right|$>0.80.

**2. The description of developed binary linear programming (BLP) approach**

For inferring the cell-specific pathways, we optimize two objective functions. The first objective function is formed aiming to minimize the difference between predicted values and measured values at the time point $t+1$in the mid-stage (Eq. (5)):

(5)

The absolute value is reformulated as . It can be verified as follow:

1. If

=$x_{j}^{k} (t+1)+(1-2x_{j}^{k} (t+1))\times0$=$x_{j}^{k} (t+1)$=

1. If

=$x_{j}^{k} (t+1)+(1-2x_{j}^{k} (t+1))\times1$=$\left|1-x_{j}^{k} (t+1)\right|$=

Therefore, Eq. (5) is equivalent to:

Given normalized data of P100, all the measured values of $x_{j}^{k} (t+1)$are constant, so the first objective function can be simplified as Eq. (6).

(6)

The second objective is to minimize the number of reactions, so that the inferred specific pathway is as smaller as possible. Therefore, we use a linear solution to simultaneously optimize above two objectives as Eq. (7):

(7)

In Eq. (7), $\gamma$is a positive value to balance two objective functions, so that the inferred cell-specific pathway will keep a high data-fitting with P100 (**Figure S3**).

The binary linear constraint set in our BLP system can be summarized as:

$z_{i}^{k} \leq x_{j}^{k} (t), i=1,...,n_{r} , k=1,...,n_{e} , j\in R_{i} .$ (8)

$z_{i}^{k} \leq1-x_{j}^{k} (t), i=1,...,n_{r} , k=1,...,n_{e} , j\in I_{i} .$ (9)

(10)

$x_{j}^{k} (t)\geq z_{i}^{k} , i=1,...,n_{r} , k=1,...,n_{e} , j\in P_{i} .$ (11)

(12)

$x_{j}^{k} (t)\leq1-z_{i}^{k} , i=1,...,n_{r} , k=1,...,n_{e} , j\in P_{i} .$ (13)

(14)

$x_{j}^{k} (t)+x_{q}^{k} (t)\geq2z_{i}^{k} , i=1,...,n_{r} ,k=1,...,n_{e} ,j\in P_{i} ,q\in R_{i} .$ (15) (16) $x_{j}^{k} (q)=0,{} k=1,2,...,n_{e} ,q\in\{t,t+1\},j\in M^{k,0} .$ (17)

$x_{j}^{k} (q)=1, k=1,2,...,n_{e} ,q\in\{t,t+1\},j\in M^{k,1} .$ (18)

$x_{j}^{k} (t)\geq x_{j}^{k} (t+1), k=1,...,n_{e} ,j\in M^{k,2} .$ (19)

$x_{j}^{k} (t)=x_{j}^{k} (t+1), k=1,...,n_{e} ,j\notin M^{k,2} .$ (20)

$x_{j}^{k} (t)=0, k=1,...,n_{e} ,x_{j} \in TS_{k} .$ (21)

$x_{j}^{k} (t)=1, k=1,...,n_{e}$ (22)

(23)

Comparing to [[2](#_ENREF_2)], the constraints (8)-(16) in our BLP approach can handle four types of linking patterns which represent the relationships between upstream proteins and corresponding downstream products in pathway topological structure, which are applied to strictly constrain the states of species and reactions (**Figure 3** and **Table 2**). Now, we introduce the meaning of the constraints (8)-(16), which are also listed in **Table 2**:

(A) **Figure 3A** suggests a single activation is presented by an “AND” gate [[2](#_ENREF_2),[3](#_ENREF_3)]. The constraints (8) and (9) means this reaction will not take place if some reagents are inactivated or the inhibitors are activated. The constraint (10) enforces that if all regents and no inhibitors are activated, then the reaction will take place. The constraint (11) ensures that a product will be activated if some reactions which it is a product occurs. In contrast, the constraint (12) means that a product will be inhibited (inactivated) if all the reactions which it appears as a product do not occur.

(B) Comparing **Figure 3B** with **Figure 3A**, the difference is that the former indicates multiple reactions with single type (activation) and the relationship between them operated by an “OR” gate; but the latter just represents a single reaction (activation). **Figure 3B** suggests there is no inhibitor in such structure, so that a reaction will take place if the reagent is activated. The product will be inhibited if all the corresponding upstream proteins are blocked. Therefore, combination of constraints (8) (10) (11) (12) could handle this kind of linking pattern in the pathway topological structure.

(C) As to **Figure 3C**, multiple inhibitions were connected with “OR” gate. An inhibition is blocked if the upstream protein is inactivated (constraint (8)). The constraint (10) means an inhibited reaction will take place if the regent is activated. Constraint (13) ensures a product is inhibited if at least one inhibition occurs. In contrast, the constraint (14) enforce that a product is activated if all the inhibitions do not occur.

(D) **Figure 3D** indicates which reactions will take place depending on both the states of reagents and product. A product can be activated by some reagents and be inhibited by other reagents. For each reaction in this topology, constraints (8), (14) and (16) should be firstly ensured. And then, constraint (15) and (13) are used to constrain activated reactions and inhibited reactions, respectively. Therefore, the states of all the species and products involved in this structure collectively determine which reactions will occur.

In addition, the states of all the variables in $X$(all the proteins) should meet the constraints (17) and (18). The constraints (19) and (20) simulate the change of phosphoprotein’s states from time point $t$ to $t+1$. If the phosphorylation level of a species is measured at time point $t$, we assume its activity may be consistent or degraded (constraint (19)). If the species is not measured, we keep its activity no change because there is no available information about it (constraint (20)). The constraint (21) set the states of some potential target proteins which are validated in literature. The constraint (22) set the states of some important activated proteins (co-factors) which are validated in literature. The formula (23) restricts the values of two groups of binary variable $X$and $Z$.

**3. The difference between BLP approach and other approach**

Mitsos et al provided two types of linking patterns, i.e., “AND” gate for single activation in **Figure 3A** and “OR” gate for multiple activations in **Figure 3B [**[**2**](#_ENREF_2)**]**, they do not present all connections in pathway topology because of the complicated pathways in KEGG and IPA database. Here we take two cases obtained from IPA database as an example (**Figure S5**). These two cases could not be represented by the patterns in **Figures 3A** and **3B** because ERK is inhibited by PAC1 or MKP, and meanwhile is promoted by MEK in **Figure S5A** here. This linking pattern is a case of mixed reactions in **Figure 3D**. Similarly, AKT and p70S6K both inhibited BAD in **Figure S5B**, which is a case of multiple inhibitions in **Figure 3C.** The four types of linking patterns in our study are frequently appeared in most of the pathways, and the four linking patterns can represent much more complicated pathway topologies.

Furthermore, our three patterns in **Figure 3(B)-(D)** can be represented by Boolean functions. Because the reactions connected with the same downstream protein are logic “OR”, the states of each reaction in **Figure 3(B)-(D)** can be represented by a Boolean function with the states of its upstream and downstream proteins. For example, the topology pattern “multiple activations” was represented in **Figure S6**. The activation (reaction) $z_{ij}$(j=1, 2, 3) is related to the input node $x_{\mathrm{ij}}$ and the output node $x_{p}$ . First, we designed a “Truth Table” as follows:

| $x_{ij}$ | $x_{p}$ | $z_{ij}$ |
| --- | --- | --- |
| 1 | 1 | 1 |
| 1 | 0 | 0 |
| 0 | 1 | 0 |
| 0 | 0 | 1 |

Obviously, an activation (reaction) in the topological patterns with “OR” gate can be represented as:$z_{ij} =\overline{x_{ij} \oplus x_{p} }=x_{p} x_{ij} +(1-x_{p} )(1-x_{ij} )=1-x_{p} -x_{ij} +2x_{p} x_{ij}$

Similarity, an inhibition (reaction) in the topological patterns with “OR” gate can be represented as:

$$z_{ij} =x_{ij} \oplus x_{p} =x_{ij} (1-x_{p} )+(1-x_{ij} )x_{p} =x_{p} +x_{ij} -2x_{p} x_{ij}$$

Above two equations can be converted as quadratic constraints; our current Boolean linear constraints are not able to represent the quadratic constraints.

Moreover, it is hard to build Boolean function based on the pattern with “AND” gate for single activation. Although this type of pattern indicates single reaction, the state of reaction depends on all the upstream proteins. If the number of upstream proteins is large, the “truth table” might be very complex making the Boolean function hard to construct.

**4. Effects of BASC normalization algorithm on resulting networks**

The BASC approach tries to find a robust threshold for data binarization by assessing whether such a threshold is possible to divide the data into two stable groups at different scales. The basic idea is to build a family of 1-dementional time series by approximating the original ordered time series gene expression data with step functions whose number of discontinuities decreases gradually. The authors proposed two algorithms (BASC A and BASC B) for implementing this idea. To detect the strongest discontinuities from the original ordered time series, BASC A calculates optimal step functions with fewer discontinuities by minimizing the Euclidean distance between the initial step function and the step functions from fine scales to coarse scales. Similarly, BASC B uses discrete scale space representations of the original step functions and calculates the step functions by defining coarse and fine scales with the amount of smoothing. Although two approaches BASC A and BASC B are different in the way of calculating step functions, BASC A performs better than BASC B without elimination of noisy genes. Hence, we applied BASC A approach to our MCF7 phosphoproteomics as following steps: (A) Here, we also use the MCF7 generic pathway map in **Figure 2A**; (B). The BASC A approach was used to binarize the p100 phosphoproteomics data of measured proteins in the generic pathway map; (C) Without elimination of measured proteins, we use our BLP model to fit the binarized data and infer the MCF7-specific pathway network.

**Figure S7** presents the MCF7-specific pathway network inferred using BASC A approach. After comparing with the pathway network inferred using our normalization method in **Figure 2B**, we found that these two cell-specific pathway networks were identical except an extra link (HDAC1--|p53) found by BASC A. Biologically HDAC1 can increase inhibition of phosphorylated active p53 [[4](#_ENREF_4)]. In addition, the fitting accuracy of our method is also similar to BASC A (**Figure S8**). In conclusion, BASC algorithm also can be applied to binarize our p100 phosphoproteomics data.

In addition, we consider that the normalization methods should be applied in a biological context; otherwise, they might induce some biases in biological meaning. For example, we have an original series $u$ about the expression (log2 treatment to control) of protein mTORC2 under all the 15 conditions:

$u$ = [-0.0714,0.1097,0.3615,-0.3903,0.1488, -0.1690, -0.3814, 0.0161, -0.0302, -2.2230, 0.2708, -0.5339, 0.2522, -0.1539, -2.7078].

According to BASC A algorithm, we calculated the vector of positions of the strongest discontinuities v=(2,2,2,2,2,2,2,2,2,2,2,2,2), which means the second value in the ordered original vector is the threshold. Thus, we obtained the binarized vector $\check{u}=[1,1,1,1,1,1,1,1,1,0,1,1,1,1,0]$. Obviously, the result indicates that some of binarized values are unreliable in biological meaning. For example, the value -0.5339 in the original series was normalized as 1 (“activated”), however, this protein under that condition should been as inactivation (0) because its expression was obviously decreased relative to control (the ratio of treatment to control is calculated as 2^(-0.5339)=0.691).

**Supplementary Figures**


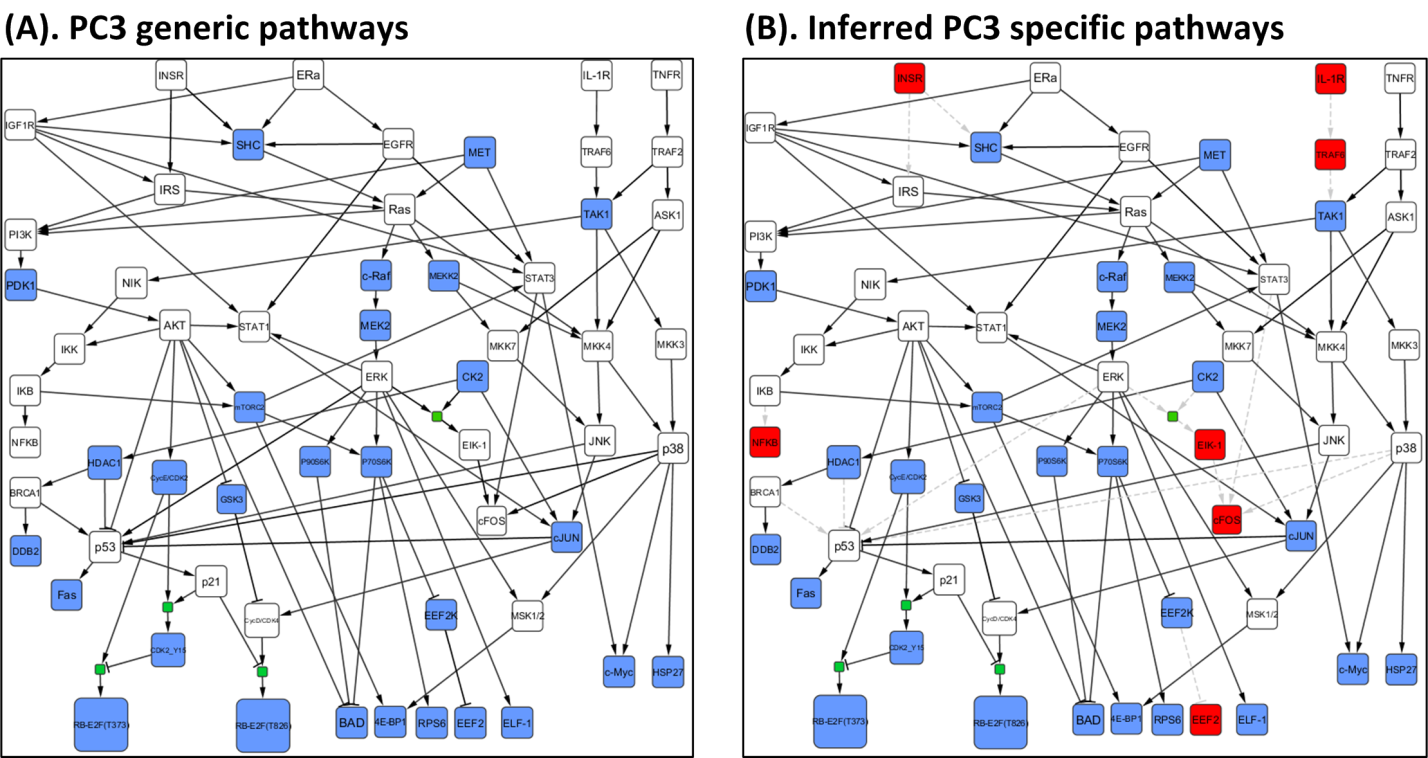


**Figure S1.** The generic pathways and inferred specific pathways of PC3 cell line. Considering the generic pathways of MCF7 (**Figure 2**) covered several classic signaling pathways, we also employ this generic pathway map to validate our proposed approach on PC3 cell line. (A) The generic pathway maps of PC3 cell line; (B) The PC3 specific pathways inferred by BLP. All the red nodes and dotted line are removed from the generic pathways.


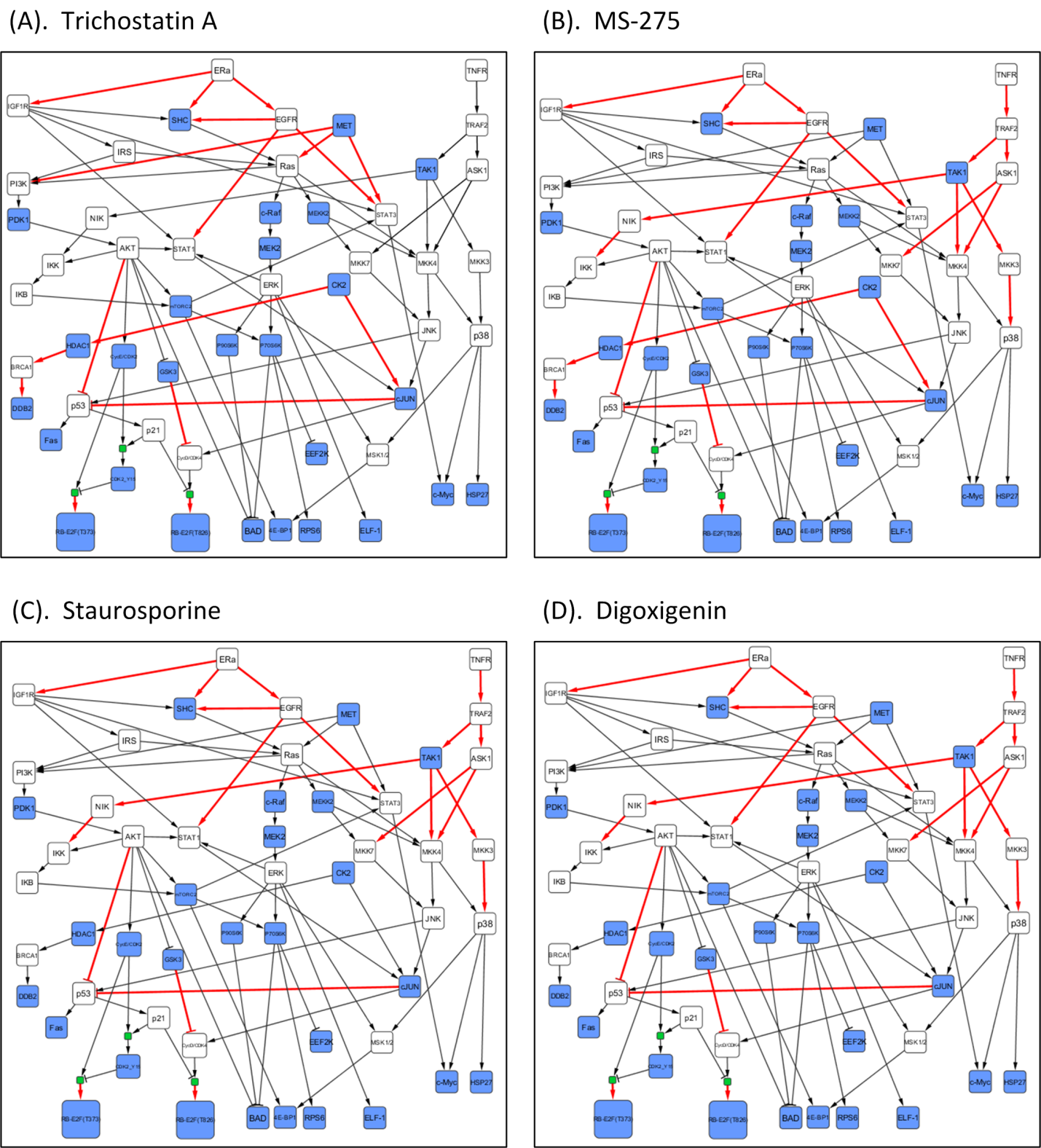


**Figure S2.** Compound-induced pathway topological alterations on PC3 cell line. (A-D) Red arrows denote the reactions are removed from the PC3 specific pathway topology by BLP in order to fit the P100 data. It also means compounds induce blocking of some reactions (red arrows) after treatment.


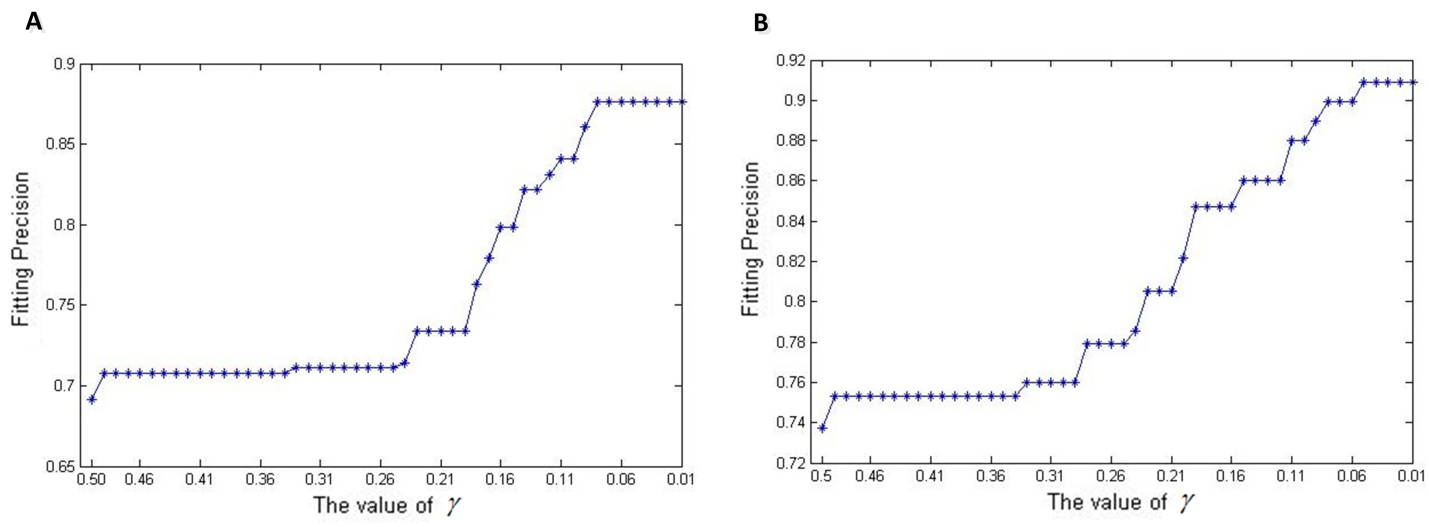


**Figure S3.** The fitting precision curves of the cell-specific pathways. (A) The fitting precision curve of MCF7-specific pathways. (B) The fitting precision curve of PC3 specific pathways. We set the values of $\gamma$ were 0.08 and 0.05 to get the optimal MCF7 and PC3 specific pathways, respectively.


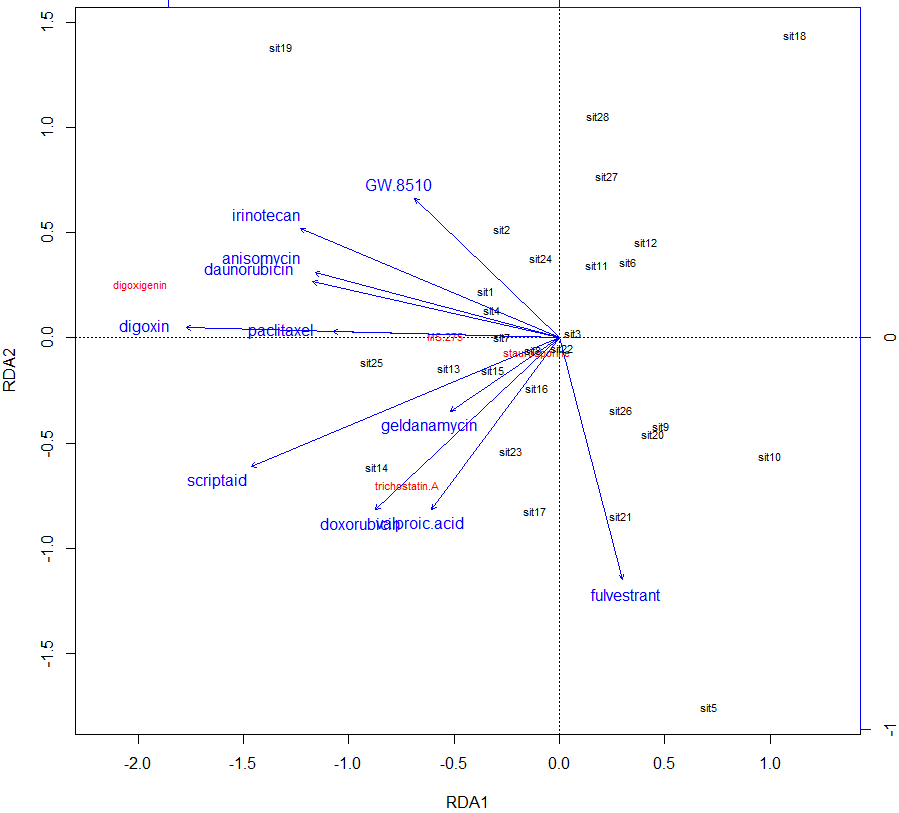


**Figure S4.** The result of redundancy analysis on P100 data with R-package “Vegan”. The blue edges denote the 11 training compounds and the red markers indicate the 4 testing compounds. "sit1”-“sit28” means the 28 measured proteins in the generic pathway map.

*
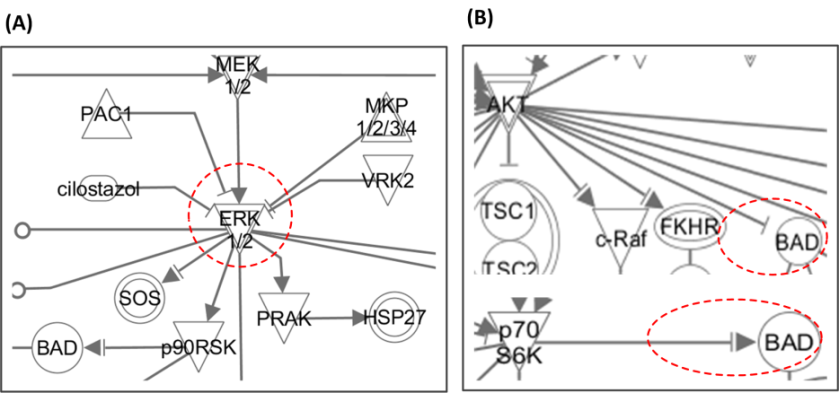
*

**Figure S5.** Two cases about two linking patterns which are mentioned **in Figure 3C-3D**.


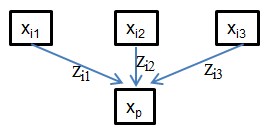


**Figure S6.** A case about multiple activations is used to describe how the Boolean function can be built depends on topological structure.

**
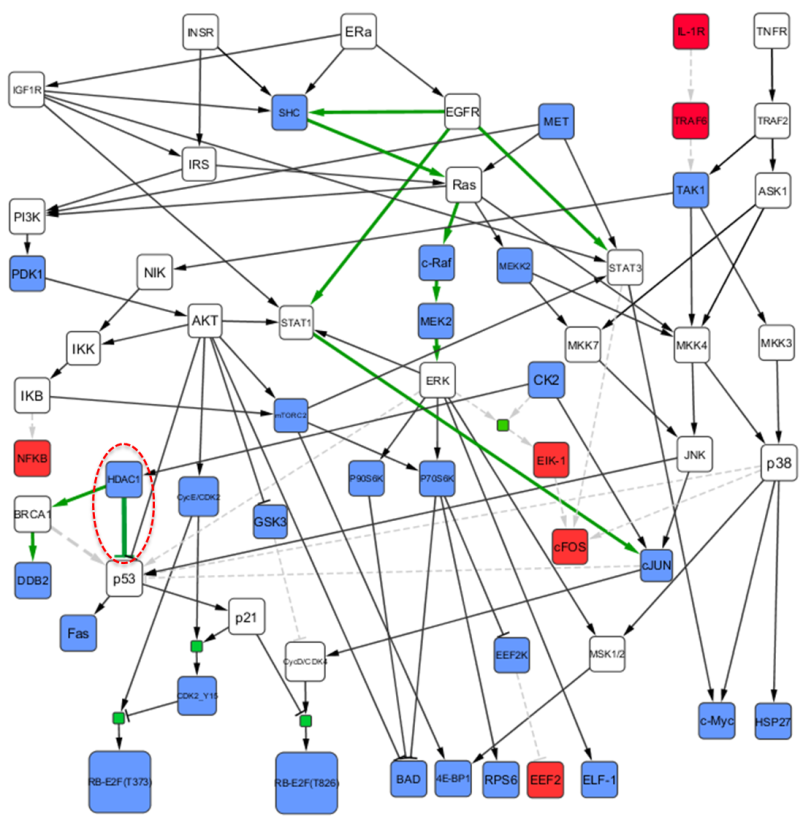
**

**Figure S7.** The inferred MCF7-specific pathway map based on the P100 data which was binarlized with **BASC_A** algorithm. The topology of this pathway map is similar to **Figure 2B**.

**Figure S8.** The performance of BASC A approach and our method.

**Supplementary Tables:**

**Table S1.** The inferred potential targets of some compounds

| **Compounds** | **Inferred target** | **Connectivity Score** | **Annotation** |
| --- | --- | --- | --- |
| Doxorubicin | JUN | 0.81851 | Doxorubicin induced a fragmentation of DNA [[5](#_ENREF_5)] |
| Daunorubicin | EGFR | 0.84296 |  |
| Irinetecan | HDAC1 | -0.97890 |  |
| Digoxin | STAT1 | 0.93775 | Digoxin induced the inhibition of cell proliferation [[6](#_ENREF_6)] |

**Table S2.** The potential downstream pathways of inferred targets included in the generic pathways.

| **Compounds** | **Inferred target** | **Downstream pathways** | **PPI interactions from HPRD** |
| --- | --- | --- | --- |
| Doxorubicin | JUN | JUN |  |
| Daunorubicin | EGFR | EGFR$\to$STAT1  EGFR$\to$STAT3  EGFR$\to\ldots\to$MEK$\to$ERK | (1) EGFR--STAT1 [[7](#_ENREF_7)]  (2) EGFR--STAT3 [[7](#_ENREF_7)]  (3) EGFR--MEK1 [[8](#_ENREF_8)]  (4) EGFR--ERK2 [[9](#_ENREF_9)] |
| Irinetecan | HDAC1 | HDAC1$\to$BRCA1$\to$P53  HDAC1$\to$BRCA1$\to$DDB2  HDAC1$\to$P53 | (1) HDAC1--BRCA1 [[10](#_ENREF_10)]  (2) HDAC1--DDB2 [[11](#_ENREF_11)]  (3) HDAC1--P53 [[12](#_ENREF_12)]  (4) BRCA1--P53 [[13](#_ENREF_13)] |
| Digoxin | STAT1 | STAT1$\to$JUN | STAT1—JUN [[14](#_ENREF_14)] |

**References**

1. Subramanian A, Tamayo P, Mootha VK, Mukherjee S, Ebert BL, et al. (2005) Gene set enrichment analysis: a knowledge-based approach for interpreting genome-wide expression profiles. Proc Natl Acad Sci U S A 102: 15545-15550.

2. Mitsos A, Melas IN, Siminelakis P, Chairakaki AD, Saez-Rodriguez J, et al. (2009) Identifying Drug Effects via Pathway Alterations using an Integer Linear Programming Optimization Formulation on Phosphoproteomic Data. Plos Computational Biology 5.

3. Saez-Rodriguez J, Alexopoulos LG, Epperlein J, Samaga R, Lauffenburger DA, et al. (2009) Discrete logic modelling as a means to link protein signalling networks with functional analysis of mammalian signal transduction. Molecular Systems Biology 5.

4. Ito A, Kawaguchi Y, Lai CH, Kovacs JJ, Higashimoto Y, et al. (2002) MDM2-HDAC1-mediated deacetylation of p53 is required for its degradation. Embo Journal 21: 6236-6245.

5. Pourquier P, Montaudon D, Huet S, Larrue A, Clary A, et al. (1998) Doxorubicin-induced alterations of c-myc and c-jun gene expression in rat glioblastoma cells: role of c-jun in drug resistance and cell death. Biochem Pharmacol 55: 1963-1971.

6. Bielawski K, Winnicka K, Bielawska A (2006) Inhibition of DNA topoisomerases I and II, and growth inhibition of breast cancer MCF-7 cells by ouabain, digoxin and proscillaridin A. Biol Pharm Bull 29: 1493-1497.

7. Xia L, Wang LJ, Chung AS, Ivanov SS, Ling MY, et al. (2002) Identification of both positive and negative domains within the epidermal growth factor receptor COOH-terminal region for signal transducer and activator of transcription (STAT) activation. Journal of Biological Chemistry 277: 30716-30723.

8. Habib AA, Chun SJ, Neel BG, Vartanian T (2003) Increased expression of epidermal growth factor receptor induces sequestration of extracellular signal-related kinases and selective attenuation of specific epidermal growth factor-mediated signal transduction pathways. Mol Cancer Res 1: 219-233.

9. Robinson FL, Whitehurst AW, Raman M, Cobb MH (2002) Identification of novel point mutations in ERK2 that selectively disrupt binding to MEK1. Journal of Biological Chemistry 277: 14844-14852.

10. Yarden RI, Brody LC (1999) BRCA1 interacts with components of the histone deacetylase complex. Proc Natl Acad Sci U S A 96: 4983-4988.

11. Taunton J, Hassig CA, Schreiber SL (1996) A mammalian histone deacetylase related to the yeast transcriptional regulator Rpd3p. Science 272: 408-411.

12. Juan LJ, Shia WJ, Chen MH, Yang WM, Seto E, et al. (2000) Histone deacetylases specifically down-regulate p53-dependent gene activation. J Biol Chem 275: 20436-20443.

13. Ouchi T, Monteiro AN, August A, Aaronson SA, Hanafusa H (1998) BRCA1 regulates p53-dependent gene expression. Proc Natl Acad Sci U S A 95: 2302-2306.

14. Zhang XK, Wrzeszczynska MH, Horvath CM, Darnell JE (1999) Interacting regions in Stat3 and c-Jun that participate in cooperative transcriptional activation. Molecular and Cellular Biology 19: 7138-7146.
